# Retraction: Samaha et al. Effects of a Single Dose of Ivermectin on Viral and Clinical Outcomes in Asymptomatic SARS-CoV-2 Infected Subjects: A Pilot Clinical Trial in Lebanon. *Viruses* 2021, *13*, 989

**DOI:** 10.3390/v13112154

**Published:** 2021-10-26

**Authors:** Ali A. Samaha, Hussein Mouawia, Mirna Fawaz, Hamad Hassan, Ali Salami, Ali Al Bazzal, Hamid Bou Saab, Mohamed Al-Wakeel, Ahmad Alsaabi, Mohamad Chouman, Mahmoud Al Moussawi, Hassan Ayoub, Ali Raad, Ola Hajjeh, Ali H. Eid, Houssam Raad

**Affiliations:** 1 Faculty of Public Health, Lebanese University, Beirut, Lebanon; ali.samaha@liu.edu.lb (A.A.S.); houssein.mouawia@ul.edu.lb (H.M.); hamad.hassan@ul.edu.lb (H.H.); ali.albazzal@gmail.com (A.A.B.); doctorchouman@gmail.com (M.C.); dr.raad.ali@hotmail.com (A.R.); hajjehpharmacy@hotmail.com (O.H.); 2 Nursing Department, Faculty of Health Sciences, Beirut Arab University, Beirut, Mazraa 1105, Lebanon; mirna.fawaz@bau.edu.lb; 3 Department of Biomedical Sciences, Lebanese International University, Beirut, Mazraa 1105, Lebanon; 4 Department of Cardiology, Rayak University Hospital, Bekaa 1801, Lebanon; ayoub_hassan@yahoo.com; 5 Ministry of Health, Beirut, Lebanon; 6 Department of Mathematics, Faculty of Sciences, Lebanese University, Nabatieh 1700, Lebanon; a.salami@ul.edu.lb; 7 Faculty of Sciences, Lebanese University, Zahle 1801, Lebanon; h.bousab@ul.edu.lb; 8 Karbala Health Directory, Baghdad 10081, Iraq; mohammed.alwakeel1983@gmail.com; 9 Department of Biology, Lille University, 59160 Lille, France; ahmad_alsaabi@hotmail.com; 10 Faculty of Nursing Sciences, Islamic University of Lebanon, Baalbek 1800, Lebanon; dr.Mmouss@yahoo.com; 11 Department of Basic Medical Sciences, College of Medicine, QU Health, Qatar University, Doha, Qatar; 12 Biomedical and Pharmaceutical Unit, QU Health, Qatar University, Doha, Qatar

The journal retracts the article, Effects of a Single Dose of Ivermectin on Viral and Clinical Outcomes in Asymptomatic SARS-CoV-2 Infected Subjects: A Pilot Clinical Trial in Lebanon [1], cited above.

Following publication, the authors contacted the editorial office regarding an error between files used for the statistical analysis.

Adhering to our complaints procedure, an investigation was conducted that confirmed the error reported by the authors.

This retraction was approved by the Editor in Chief of the journal.

The authors agreed to this retraction.
